# Supplementary material for: Controlling the shape of 3D microstructures by temperature and light
Source: Nat Commun. 2019 Jan 16;10:232. doi: 10.1038/s41467-018-08175-w (PMC6335428; doi:10.1038/s41467-018-08175-w)
Supplement: Supplementary file 1 — Supplementary Information [file 41467_2018_8175_MOESM1_ESM.pdf]

Supplementary Information

Controlling the Shape of 3D Microstructures by Temperature and Light

Marc Hippler, Eva Blasco, Jingyuan Qu, Motomu Tanaka, Christopher Barner-Kowollik, Martin Wegener, and Martin Bastmeyer**

Supplementary Figures

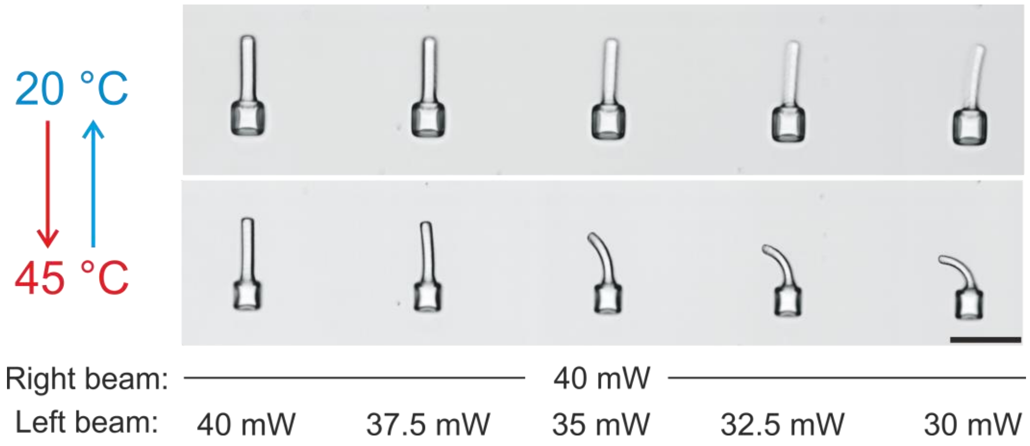

Supplementary Figure 1. Temperature response as a function of local exposure dose. The structures are designed as simple bi-material beams with varying local exposure doses (see annotation on bottom). With increasing difference between left and right beam, the bending to the left-hand side gradually increases. Scale bar is 30 µm.

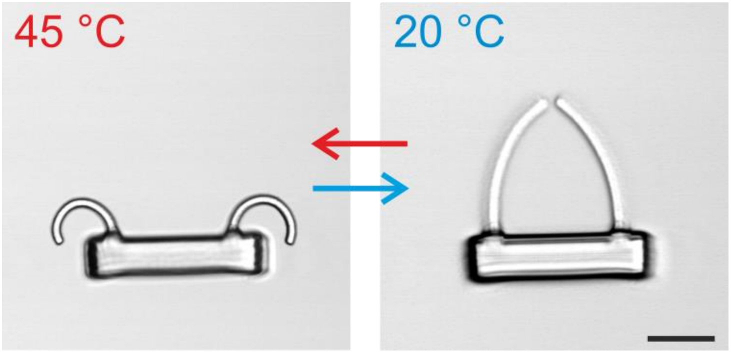

Supplementary Figure 2. Operation of a bi-material gripper. The gripper consists of two bi-material beams with the less crosslinked part at the outer side. In this configuration, the gripper is closed at room temperature and opened at elevated temperature. Scale bar is 20 µm.

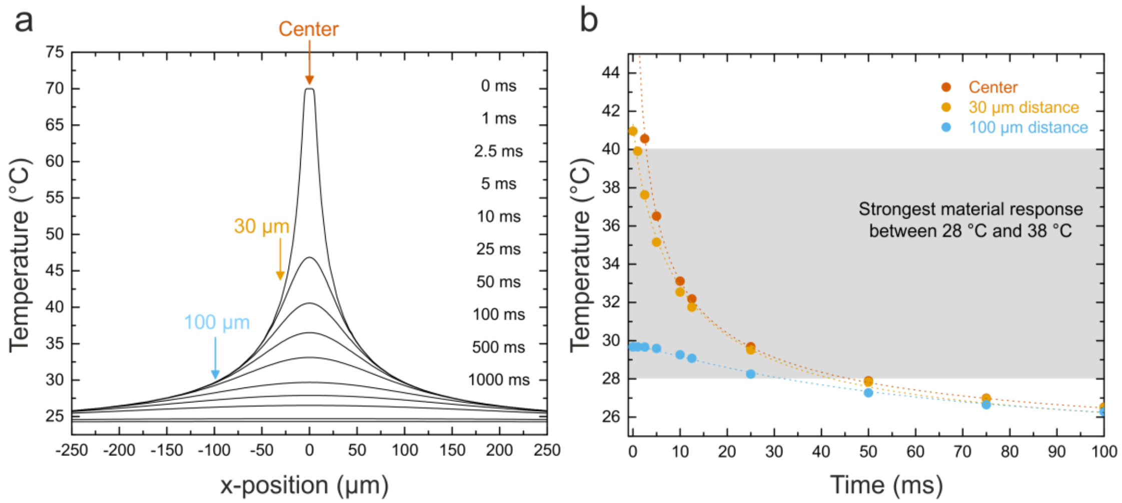

Supplementary Figure 3. Calculation of heat diffusion. a) Cuts through the temperature profile in the water bath for different time points after switching off the light. b) Temperature as a function of time for three specific distances from the central base. The grey area marks the nonlinear response regime of the material (see Figure 3b).
