# Supplementary material for: Controlling the shape of 3D microstructures by temperature and light
Source: Nat Commun. 2019 Jan 16;10:232. doi: 10.1038/s41467-018-08175-w (PMC6335428; doi:10.1038/s41467-018-08175-w)
Supplement: Supplementary file 3 — Description of Additional Supplementary Files [file 41467_2018_8175_MOESM3_ESM.pdf]

Description of Additional Supplementary Files

File Name: Supplementary Movie 1
Description: Image stack of optical micrographs of a hetero-microstructure, taken at different temperatures between 20 °C and 50 °C.

File Name: Supplementary Movie 2
Description: Bright-field optical movie of 3x3 nominally identical hetero-microstructures. The temperature is increased from 20 °C to 45 °C. Playback speed was increased by factor of five.

File Name: Supplementary Movie 3
Description: Bright-field optical movie of four hetero-microstructures with lengths from 30 µm to 60 µm. The temperature is increased from 20 °C to 45 °C. Playback speed was increased by factor of five.

File Name: Supplementary Movie 4
Description: Bright-field optical movie of a hetero-microstructure with beam length of 120 µm. The temperature is decreased from 45 °C to 20 °C. Playback speed was increased by factor of five.

File Name: Supplementary Movie 5
Description: Bright-field optical movie of a hetero-microstructure with two alternating material segments. The temperature is increased from 20 °C to 45 °C. Playback speed was increased by factor of five.

File Name: Supplementary Movie 6
Description: Bright-field optical movie of a hetero-microstructure with three alternating material segments. The temperature is increased from 20 °C to 45 °C. Playback speed was increased by factor of five.

File Name: Supplementary Movie 7
Description: Bright-field optical movie of four hetero-microstructure with a distance of 100 µm. The laser spot is focused on the post of the second structure and the shutter opened and closed several times.

File Name: Supplementary Movie 8
Description: Sequence of bright-field optical movies of a single hetero-microstructure. The exposure and settling time of the focused laser spot is changed between the movies to generate different actuation responses.
